# The association of BMI and knee pain among persons with radiographic knee osteoarthritis: A cross-sectional study

**DOI:** 10.1186/1471-2474-9-163

**Published:** 2008-12-12

**Authors:** Matthew W Rogers, Frances V Wilder

**Affiliations:** 1 The Arthritis Research Institute of America, 300 S. Duncan Avenue, Suite 188, Clearwater, Florida 33755, USA

## Abstract

**Background:**

Many people with radiographic knee osteoarthritis (RKOA) do not present with pain. It is suspected that such persons tend toward lower body mass index (BMI). The purpose of the study was to explore the relationship between BMI and knee pain among persons with RKOA.

**Methods:**

Subjects in the Clearwater Osteoarthritis Study with RKOA (N = 576) were classified as reporting knee pain (Pain) or no knee pain (No Pain). WHO-classified BMI categories were compared by pain status. Odds ratios were calculated for the four elevated BMI groups, with the normal BMI group as the reference group. Elevated BMI was the risk factor, and knee pain status was the outcome factor.

**Results:**

Pain subjects presented with a higher mean BMI (30.4 kg/m^2^) compared with No Pain subjects (27.5 kg/m^2^) (p < 0.0001). Unadjusted and adjusted odds ratios demonstrated a positive association between BMI group and pain for each successive elevated BMI category. Adjusted odds ratios ranged from 1.6 for the Pre-obese group (p < 0.05) to 7.5 for the Obese III group (p < 0.0001).

**Conclusion:**

Among subjects with RKOA, those presenting with an elevated BMI had a greater likelihood of knee pain compared to subjects with a normal BMI, and this chance rose with each successive elevated BMI category. As BMI is a modifiable risk factor, longitudinal research is needed to confirm these findings and elucidate the mechanisms underlying this relationship.

## Background

Osteoarthritis (OA) of the knee is particularly common in older adults, with one survey finding radiographic evidence of knee OA in 33% of subjects aged 63 – 93 years [1]. Symptomatic knee OA is a major cause of physical disability afflicting older persons, due to pain, stiffness, and joint instability [2,3]. While the cause of radiographic knee OA (RKOA) remains unclear, it has been associated with various risk factors, such as advancing age, female gender, genetic predisposition, prior knee injury, certain occupations, biomechanical gait and alignment defects, and obesity. Of these, obesity is perhaps the most important risk factor associated with the incidence of RKOA [4-8].

Epidemiological studies have linked RKOA-related knee pain with increasing radiographic severity, bone marrow lesions, bone ulceration, quadriceps weakness, and psychological factors [9]. However, a clear profile of those who experience pain and those who do not remains to be elucidated. Independent of radiographic features, many patients with RKOA do not present with accompanying pain [9,10]. Careful study of these knee pain-free RKOA patients may help elucidate both causes and potential palliative treatments for those with painful RKOA. As there is no cure for OA on the horizon, it is important to identify the factors that influence the risk of symptomatic knee OA, as this could help clinicians guide their patients in taking steps (e.g. weight loss) to prevent or delay the onset of pain and impairments that may lead to work disability, daily living disability, and joint replacement surgery.

It is known that persons with higher body mass indexes (BMI; kg/m^2^) are more likely than persons with normal BMI to report idiopathic knee pain and accompanying disability [11-13]. Although this would suggest that elevated BMI is a potential risk factor for knee pain among persons with RKOA, relatively little research has been conducted in this area. In 2007, Marks [14] reported that among individuals with RKOA, those with higher BMIs reported more pain. Similarly, Felson and colleagues [15] found that study subjects with symptomatic knee OA presented with elevated BMI levels compared to their asymptomatic counterparts. However, uncertainty remains regarding this potential relationship. A 1995 study investigated the relationship between pain and BMI among patients with RKOA and found no association [16]. Our current study aims to help clarify these findings by reporting odds ratios to further quantify the relationship between pain and BMI among persons with RKOA. Using a sample of subjects with RKOA, our cross-sectional study [17] seeks to quantify the association between BMI and knee pain among subjects with radiographic evidence of knee OA.

## Methods

The Clearwater Osteoarthritis Study (COS) is an on-going community-based cohort study designed to identify the major risk factors for the development and progression of OA. A complete description of the COS has been previously published [18]. In brief, over 3700 people have been enrolled in the study to date, which began in 1988 and will be conducted over a 25-year period. The COS was approved by the community-based Institutional Review Board of the Arthritis Research Institute of America, an uncompensated, non-employee board that has representation from the medical disciplines. Subjects are recruited by community announcements and seminars at community organizations, as well as through the local school system and government employees. Subjects sign informed consent documents at each exam. Persons of both genders age 40 years and older, with or without OA, are included. The exclusion criteria include self-reported rheumatic disease (other than OA), disabling neuralgic disease, wheelchair dependence, or mental incompetence. After eligibility is determined and informed consent has been obtained, the subject's height and weight is measured without shoes. Subjects complete a questionnaire detailing demographics, family and medical history, work and lifestyle history, a self-reported functional assessment, and a module describing joint symptoms. Radiographs, including a weight-bearing anteroposterior view of both knees, are taken at the first and all subsequent exams.

### Subjects

**Figure 1:**
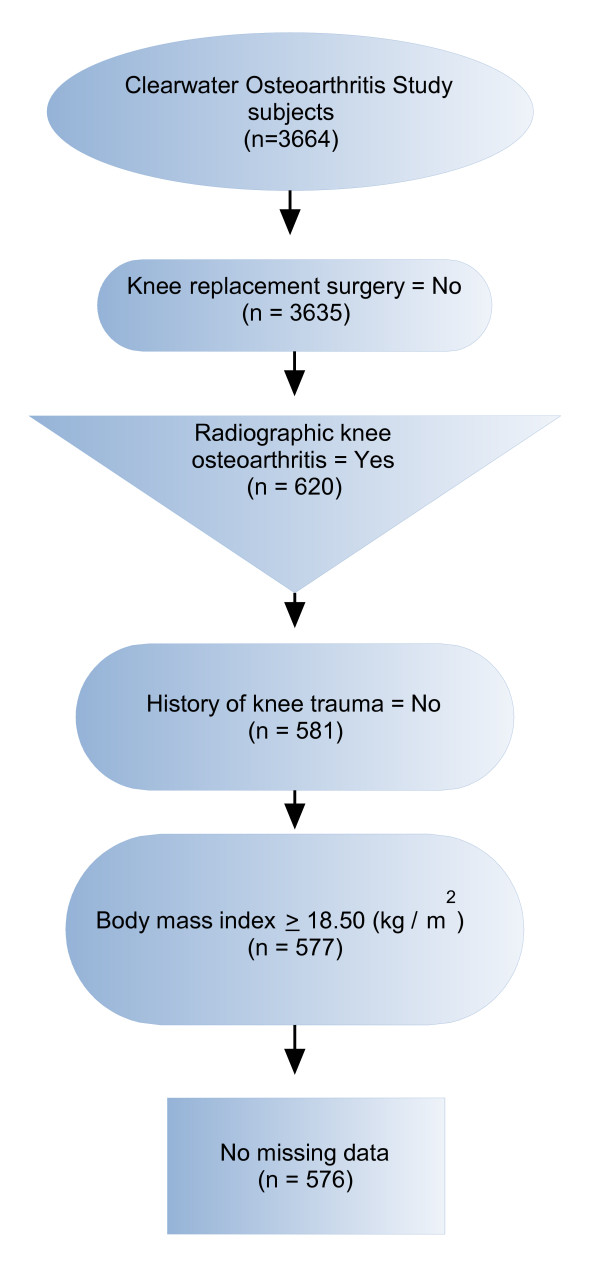
Sample selection from the Clearwater Osteoarthritis Study.

Our primary null hypothesis for the present study was that, among subjects with RKOA, elevated BMI is not associated with pain status. We used a cross-sectional design, and our study sample included 576 subjects. Sample selection from the COS is detailed in Figure 1. COS subjects who had undergone partial or total knee joint replacement surgery or reported a history of knee trauma were excluded from the analysis. RKOA was determined by the criteria of Kellgren & Lawrence [19] and defined as Grade 2 or higher readings in either or both knees. All radiographs were graded by a board certified radiologist. Positive history of knee injury was determined by affirmative responses to either of the following questions: *"Have you ever had a fractured knee?*" or, *"Have you ever had a severe twisting of either knee with resultant sprain or swelling lasting more than two weeks?" *Subjects with knee injury history were excluded, so our sample subjects would more closely align with the American College of Rheumatology criteria for primary knee OA [20]. Based on knee pain status, subjects were categorized as having knee pain (Pain) or no knee pain (No Pain). Subjects categorized as Pain must have presented with pain in (at least) the knee with radiographic evidence of OA. The questionnaire depicted a human figure on which subjects were asked to circle up to four noted areas of the knee(s) that were painful. Subjects that reported "No pain" or "Don't know" were included in the No Pain category. A pain rating scale was not provided.

Using the World Health Organization's International Classification [21], subjects were placed into one of five BMI groups (kg/m^2^) as follows: Normal (18.5 to 24.9); Overweight/Pre-obese (25 to 29.9); Obese class I (30 to 34.9); Obese class II (35 to 39.9); and Obese class III (40+). There were too few underweight subjects (BMI < 18.5; n = 3) with RKOA for meaningful analysis of that group. BMI was compared by knee pain status (Pain or No Pain). Factors thought to be potential confounders when assessing the relationship between BMI and knee pain were considered; these included disease severity (as measured by OA grade), age, gender, occupation, education, and self-reported disease status for diabetes, heart disease, and stroke. Subjects were classified as "High-risk occupation" if they reported that the job they had for most of their life involved "*standing most of the time*" or *"jolting of the feet and legs" *(Table 1). Age and gender were retained in the final adjusted analyses.

**Table 1:** Study sample demographics by pain status among subjects (N = 576) with radiographic knee osteoarthritis^a^

	Pain (N = 329)	No Pain (N = 247)
Age mean (SD)	67.1 (9.0)	66.8 (10.7)
Age minimum	40.0	40.0
Age maximum	89.0	89.0
BMI^b‡ ^mean (SD)	30.4 (6.4)	27.5 (4.9)
BMI minimum	18.9	18.9
BMI maximum	65.6	46.6
Female^† ^(%)	71.1	60.3
High-risk occupation (%)	33.1	32.4
Education (no high school) (%)	10.1	11.0
Diabetes (%)	1.2	0.8
Heart disease (%)	8.2	9.7
Stroke (%)	4.3	2.8

^a ^Kellgren and Lawrence scale for radiographic osteoarthritis (Grades 2+).

^b ^BMI = body mass index (kg/m^2^).

^† ^P < 0.01; ^‡ ^P < 0.0001

### Statistics

Frequencies, percentages, and means were used to provide descriptive data regarding the study sample. To estimate the strength of the association between BMI and knee pain, we computed odds ratios (OR) [22] using multiple logistic regression [23,24]. Knee pain status (yes/no) was the outcome factor. With BMI as the exposure factor, subjects with normal BMI (18.5 to 24.9 kg/m^2^) served as the reference group for the analyses (e.g. Normal vs. Obese III). A regression model was run for each BMI group. To address the potential for confounding when assessing the association between BMI and knee pain, we ran an adjusted statistical model (Pain = BMI + age + gender + RKOA grade). Logistic regression was used, as we had a binary outcome and several explanatory factors (e.g. gender) in the statistical model [25]. Based on preliminary analyses, as well as consideration of known factors related to OA, we built our final adjusted model with the inclusion of BMI, age, gender, and disease severity (as measured by OA grade). Confidence internals were reported for the odds ratios. Every tenth subjects' assembled radiographs were independently interpreted by a non-affiliated radiologist blinded to the results of the first reading. The inter-observer variability of x-ray interpretations was calculated using the kappa coefficient [26] measuring the amount of agreement that is above random chance. SAS/STAT^® ^software version 9.2 was used for all analyses [27].

## Results

The inter-reader reliability of x-ray interpretations between the first and second radiologist reflected 93% agreement (kappa = 0.85).

The Pain and No Pain groups included 329 and 247 subjects, respectively. Study sample demographics are presented in Table 1. BMI category and OA grade counts by pain status are presented in Table 2. Evaluation for potential confounding showed no clinically relevant differences regarding diabetes, heart disease, stroke, occupation and education between the Pain and No Pain groups.

**Table 2:** BMI category and OA grade counts, by pain status among subjects (N = 576) with radiographic knee osteoarthritis^a^

	n	Pain (N = 329)	No Pain (N = 247)
Normal BMI^b^	155	66	89
Pre-Obese	208	113	95
Obese I	119	75	44
Obese II	61	47	14
Obese III	33	28	5
			
RKOA Grade 2	331	155	176
RKOA Grade 3	150	100	50
RKOA Grade 4	95	74	21

^a ^Kellgren and Lawrence scale for radiographic osteoarthritis (Grades 2+).

^b ^BMI = body mass index (kg/m^2^) WHO Classification: Normal 18.5–24.9; Pre-Obese 25.0–29.9; Obese I 30.0–34.9; Obese II 35.0–39.9; and Obese III 40.0 +.

The Pain group presented with a significantly higher BMI (30.4 kg/m^2^) when compared with the No Pain group (27.5 kg/m^2^) (p < 0.0001).

Odds ratios for the association between BMI and knee pain are presented in Table 3. Unadjusted analyses demonstrated a positive association for this relationship. Subjects in the lowest elevated BMI category (Pre-Obese) showed the smallest association (OR = 1.6) while subjects in the highest BMI category (Obese III) showed the highest association (OR = 7.8). For the four elevated BMI groups, a positive relationship with pain was found in the unadjusted and adjusted analyses. After adjustment for age, gender, and RKOA grade, subjects in the Pre-obese category again demonstrated the smallest association (OR = 1.6) while subjects in the Obese III category demonstrated the highest association (OR = 7.5).

**Table 3:** Association between body mass index and knee pain among subjects (N = 576) with radiographic knee osteoarthritis^a^

		Unadjusted		Adjusted^d^	
BMI^b^	(n)	OR^c^	95% CI	OR	95% CI
Pre-Obese	(208)	1.6 *	1.1 – 2.4	1.6*	1.0 – 2.5
Obese I	(119)	2.3 **	1.4 – 3.8	2.0**	1.2 – 3.4
Obese II	(61)	4.5***	2.3 – 8.9	3.9**	1.8 – 8.3
Obese III	(33)	7.8 ***	2.8 – 20.6	7.5**	2.5 – 22.6

* P < 0.05; ** P < 0.01; *** P < 0.0001.

^a ^Kellgren and Lawrence scale for radiographic osteoarthritis (Grades 2+).

^b ^BMI Body Mass Index (kg/m^2^) WHO Classification: Pre-Obese 25.0–29.9; Obese I 30.0–34.9; Obese II 35.0–39.9; and Obese III 40.0 +.

^c ^OR = odds ratio. Referent group for all odds ratios is Normal BMI 18.5–24.9 (n = 155).

^d ^Adjusted odds ratio models: [Pain = BMI + age + gender + RKOA grade].

## Discussion

Our results support those of two prior studies [14,15] and are in contrast to a third [16]. However, to our knowledge, the relationship among BMI, RKOA and knee pain has not been previously reported in terms of odds ratios. Overweight and obese subjects with RKOA were found to have a higher likelihood for knee pain compared to normal weight RKOA subjects. The chance for knee pain rose substantially with each successive elevated BMI category. Those in the highest BMI category, Obese III, had the highest chance compared to subjects in the Normal BMI group.

While our results demonstrate a strong link between increased BMI and knee pain among persons with RKOA, indicating overweight and obesity as a potential cause of knee pain, the cross-sectional design limits our ability to rule out alternative explanations. One such explanation may be that knee pain precedes a high BMI. That is, RKOA-associated knee pain in a normal weight person may lead to a sedentary lifestyle which in turn leads to weight gain. High BMI in these patients would therefore be secondary to knee pain, rather than the reverse. However, other investigators have discounted this notion, concluding there is "*no evidence that the association between BMI and knee OA is stronger for those with knee symptoms" *[9]. Other studies have demonstrated that overweight and obese patients with knee pain experience a reduction in pain and improvement in physical function from weight loss [28-30]. It is therefore reasonable to speculate that pain-free RKOA patients of normal weight are at a reduced risk for developing knee pain, and could remain at this lower risk by maintaining a normal BMI.

Sharma and Kapoor [9] propose several mechanisms that could account for knee pain among persons with RKOA, including bone marrow lesions, bone ulceration, quadriceps weakness, and psychological factors. It is unclear how these factors may relate to BMI. A recent study [31] found a significant association between obesity and several psychiatric disorders, including major depression and dysthmia. Other reports have related depression to pain in general [32] and to knee pain, specifically [33]. It is reasonable to assume that persons with higher BMI may manifest greater knee OA pain at least in part due to higher rates of depression or other psychological factors.

## Conclusion

Our findings can be built upon with a longitudinal study designed to further elucidate the involved mechanisms. It might also be instructive to follow pain-free persons with RKOA (without a history of knee trauma) to observe possible associations between changes in BMI and the risk for knee pain.

## Competing interests

This research was funded by private donations. The authors declare that they have no competing interests.

## Authors' contributions

MWR developed the study concept and drafted the manuscript. FVW designed and conducted the statistical analysis and helped draft the manuscript.

## Pre-publication history

The pre-publication history for this paper can be accessed here:


